# 

**Published:** 1852-03

**Authors:** 


### CONTENTS:

### PART I—ORIGINAL COMMUNICATIONS.

ART. 1—Cerebro-Spinal Arachnitis or Paralysis, by Jas. Whitmire, M. D. - - 382
" 2—Neuralgia treated by Internal use of Chloroform, by P. A. Allaire. M. D. - 388
" 3—Cases occurring in the practice of S. W. Ritchey, M. D. - - - 392
" 4—Cases and Clinical observations in the Medical Wards of the Illinois General Hospital, by N. S. Davis, M. D. - - - - - - - - - 392
" 5—Poisoning with the Per Chloride of Mercury, by W. W. Goff, M. D. - - 406
" 6—Enlargement of one of the Ethmoidal Cells, etc. by Daniel Brainard, M. D. - 403
" 7—On the treatment of Ununited Fracture, by Daniel Brainard, M. D. - - 410
" 8—Ligature of the Formural Artery, for Popliteal Aneurism, by Daniel Brainard, M. D. 414
" 9—Aconitum Napellus, by John A. Preston, M. D. - - - - - 415
" 10—Dislocation of the Femur into the Obturator,—Reduction by a block between the thighs, after the Pulleys and Jarvis' Adjuster had failed by D. Brainard, M. D. 418
" 11—Use of Diluted Pyroligeneous Acid as a Gargle, by John Evans M. D. - 419

### PART II—REVIEWS AND NOTICES OF NEW WORKS.

ART. 1—Transactions of the Amer. Med. Association. - - - - - 421
" 2—Homœopathy: An examination of its Doctrines and Evidences, by Worthington Hooker, M. D. - - - - - - 429
" 3—Lectures on Scarlet Fever, by Caspar Morris M. D. - - - 432

### PART III—SELECTIONS.

ART. 1—Dislocation of the Femur on the Dorsum Ilii, reducible without Pulleys, or any mechanical powers: Three cases, by W. W. Reid, M. D. - - - 445
" 2—Experiments with the Ligature on Animals. by J. Piernans, M. D. - - 442
" 3—The Transfusion of Blood. - - - - - - - 444
" 4—Meeting of the Amer. Med. Association. - - - - - 445
" 5—New Theory of Respiration and Circulation; Interesting experiment on an Alligator 446
" 6—Motive power of the Blood; Experiments on an Alligator at New Orleans. 452
" 7—Prof. Dowler and the Medico Chirurgical Review. - - - 457
" 8—Welfare of the Medical Profession. - - - - 450
" 9—Homoeopathy and the Royal College of Physicians, London. - - - 460

### PART IV—EDITORIAL.

ART. 1—Valedictory. - - - - - - $46^{1}_{3}$
" 2—Electic Journal of Education and Literary Review. - - $46_{4}$
" 3—Dr. Talcott's Address. - - - - - - $46_{6}$
" 4—Transactions of the Amer. Med. Assoriation of Southern Central N. Y. - $46_{5}$
" 5—The Medical Profession and the Public Press. - - - - - - $46_{7}$
" 6—Report ot the Indiana Hospital for the Insane.for the year 1851, - - $46_{9}$
" 7—Annual Commencement of Rush Medical College. - - - - $46_{1}$
" 8—Notice to Subscriber---Extension of time. - - - - 47
" 9—Drake's Discourses. - - - - - - $47^{1}$
" 10—Miscellaneous Medical Intelligence. - - - - - $47^{2}$

